# Evaluation is Key: Providing Appropriate Evaluation Measures for Participatory and User-Centred Design Processes of Healthcare IT

**DOI:** 10.5334/ijic.5529

**Published:** 2021-06-21

**Authors:** Lorenz Harst, Bastian Wollschlaeger, Jule Birnstein, Tina Fuchs, Patrick Timpel

**Affiliations:** 1 Center for Evidence-based Healthcare, University Hospital and Faculty of Medicine Carl Gustav Carus, Technische Universität Dresden, DE; 2 Chair of Technical Information Systems, Technische Universität Dresden, DE; 3 Zittau/Görlitz University of Applied Sciences, DE; 4 Prevention and Care of Diabetes, Department of Medicine III, University Hospital and Faculty of Medicine Carl Gustav Carus, Technische Universität Dresden, DE

**Keywords:** Healthcare IT, user-centred design, implementation, evaluation, acceptance

## Abstract

**Introduction:**

The increasing availability of healthcare IT has the potential to improve the integration of health services. Existing projects developing healthcare IT mostly disregard the potential and importance of incorporating user feedback and proper evaluation measures to gain user feedback throughout the development process. We therefore provide methodological guidance for evaluation in a stepwise user-centred design process.

**Methods:**

Based on a literature review we propose adequate methods for data collection in each phase of participatory and user-centred healthcare IT development. In order to provide an orientation within the plethora of development processes used in practice, we consolidate a generic blueprint process from the literature review. The applicability of our methodological guidance is shown in three diverse use cases from the field of integrated care.

**Results:**

From 14 literature items, we identified common evaluation methods including, among others, interviews, focus groups, and surveys. These methods can be associated to six typical development phases that could be derived from research: *State of the Art Research, Requirement Analysis, Conceptual Prototype, Preliminary Prototype, Full Prototype, Full Application*. The use cases demonstrate the value of qualitative methods and mixed methods designs.

**Discussion:**

Our methodological guidance has proven applicable for designing healthcare IT solutions from scratch – both for patient and professional settings – and to develop a platform for combining existing component-based solutions. In integrated care settings, where a wide range of stakeholders with multiple needs exist, we thus provide methodological guidance on how to involve users in the development process.

**Conclusion:**

Our stepwise methodological guidance helps to design and properly evaluate healthcare IT solutions, which meet the user needs and requirements, for integrated care settings.

## Introduction

Patients with chronic conditions are in need of medical care delivered by multiple stakeholders [1]. Apart from that, they often experience a variety of competing needs, which are not limited to morbidity-related aspects, but include, e. g., the need for a social support network [2]. These needs, prioritized differently from one individual patient to another, often remain unmet, partially due to a lack of funding [3]. In response to this, targeted chronic care, e.g. individualised glycaemic targets and decision-making for patients with diabetes, is seen as a key to address these unmet needs [4].

Due to this demand for individualised care measures, chronically ill patients are the ideal target population for digital health devices, especially those that fall under the definition of telemedicine [5]. These devices can provide continuous monitoring of clinical and behavioral parameters, such as diet and physical activity [6], and, above all, individualised health care provider feedback based on the collected values [7]. As the emergence of mHealth applications allows for tailoring digital health interventions to the needs of specific patient groups [7], applying development processes that allow for continuous user participation becomes even more pressing. While, lately, the concept of patients as consumers of digital health applications has gained prominence [8], patients still differ from other technology users in several key characteristic. As the example of diabetes self-management shows, patients need to continuously monitor their blood glucose levels as well as dietary and physical activity behaviors [9]. Such, devices that aid this process have to be used continuously as well in order to be effective [10]. Discontinued use due to loss of motivation, as it can be the case with any digital device [11], therefore poses a threat to therapeutic outcomes. Furthermore, potential users, i. e. patients with chronic diseases, are mostly older, less interested in technology in general and therefore less skilled in using novel devices, and, maybe above all, used to and reliant on the personal connection to their doctor [12,13]. Fitting a digital device to the user needs becomes even more complicated when both patients and health care providers need to use the device. For instance, in diabetes care, research on patients’ and health care providers’ needs and expectations indicates significantly different priorities [3]. Furthermore, privacy concerns, which are often disregarded by users when it comes to sharing holiday pictures, amount to a usage barrier when personal health data are to be shared [14].

A recent synthesis of barriers in telemedicine implementation processes indicates that the majority of these barriers arise from a lack of theoretical underpinnings for individual end-users’ acceptance [15]. Theories of technology acceptance, which include characteristics of both users and the technology as predictors of acceptance, should be used to guide the design process [16]. Thus, the acceptance of any healthcare IT application by patients and their direct social environment, as well as by health care providers, can be ensured early within the design process. A non-consideration of acceptance and users’ preferences during the development of a health IT application may ultimately lead to non-effective applications due to low acceptance and usability rates [17]. Coming from IT development, therefore, the groundwork of Norman and Draper for user-centred design [18] is also demanded by the ISO norm 9241-210 for interactive systems [19]. A focus on perspectives of both providers and patients in the design process [20] is in line with recent calls to shift from process evaluations of integrated care solutions to a more participatory form of implementation [21] and evaluation [22], commonly called participatory design [23].

Before being applied in standard care, healthcare IT and digital devices in healthcare have to provide evidence on safety and/or the effectiveness regarding clinical or health-related outcomes. However, due to the still overarching dominance of IT experts and institutions in the development processes of such devices, both the participation of end-users (patients and providers alike) as well as evidence-based data collection using adequate methods is still lacking [8,24]. To this date, there is a number of prototypical processes for the user-centred design of specific health care IT applications, such as teleconsultation systems [25], web-based health information databases [26] and consumer mobile health applications [27]. For integrated care systems in a community setting, Wodchis et al. propose a standardized implementation framework [28], which, however, is not based on existing concepts and does not provide methods to support the intended user and provider participation.

Based on a literature review, we provide methodological guidance indicating how to collect data e. g. on users’ needs, acceptance and priorities within the relevant development phases of a generic blueprint for user-centred and participatory design procedures, which can be applied by developers of future healthcare IT applications. Based on three use cases, we then demonstrate the merits of applying some of the proposed methods in the development process of different digital health applications in integrated care settings.

## Methods

Since in practice a variety of models for user-centred and/or participatory application development are used, such as the ones proposed by Esser & Goossens [25], Arsand & Demiris [8] and Sutcliffe & colleagues [31], and no standard development model can be identified, we first consolidated a generic blueprint for these processes. The blueprint is not intended to serve as a suggestion on how to develop healthcare IT applications, but rather shall be used by developers as a common ground for choosing appropriate evaluation methods during the development process.

To this end, we conducted a literature review [29] to derive a generic blueprint for user-centred and participatory design processes, which then was used as a reference for when to apply which evaluation methods.

The review of models for healthcare IT development and evaluation was conducted within PubMed and Google scholar. The former was chosen as it is likely to include research on adequate methods for user-centred and participatory design processes. The latter was chosen to complement the search. Technical databases such as IEEEXplore were explicitly disregarded as they rather deal with research on technical feasibility and focus less on evaluation aspects.

Search terms included synonyms for user-centred design, user acceptance and the terms “framework” or “model”. Only research proposing and/or applying a complete user-centred design process was included. No limitations for publication types, domains (prevention, primary or inpatient care) or disease types addressed by the healthcare IT application in question were defined. The same applies to publication dates.

Based on the results, a generic blueprint for the user-centred and participatory design of healthcare IT applications was derived. First, the design and development steps suggested in more than one of the included references were categorized and sorted chronologically in an inductive process derived from Mayring’s qualitative content analysis. This method is based on rearranging text material in order to uncover underlying patterns [30]. In order to validate the applicability of our thus derived generic blueprint, we aligned it with existing prominent models of user-centred and/or participatory design of health care IT, such as the ones mentioned above.

The evaluation methods suggested in the included literature were matched with the phases of the generic blueprint based on suggestions made in the identified literature. The literature review, as well as the consolidation of the blueprint and the mapping of the methods, were carried out independently by two researchers in order to minimize bias.

Three use cases, one for a mHealth application for diabetes self-management, one for a digitally supported discharge management system coordinating patients’ transition and post-hospital care, and one for an automated design method for Ambient Assisted Living (AAL) solutions, will illustrate the applicability of the methods suggested in each step. Short paragraphs on lessons learned are provided to critically reflect on the applied methods and their role in the use cases.

## Results

First, a generic blueprint for user-centred and participatory design processes of healthcare IT applications is presented as a means to provide structure for associating the evaluation methods. In a second step, appropriate evaluation methods for each phase are suggested. In a final step, three different use cases are presented and discussed illustrating the applicability of the methodological guidance.

### The generic blueprint for user-centred and participatory design processes

**Figure 1:**
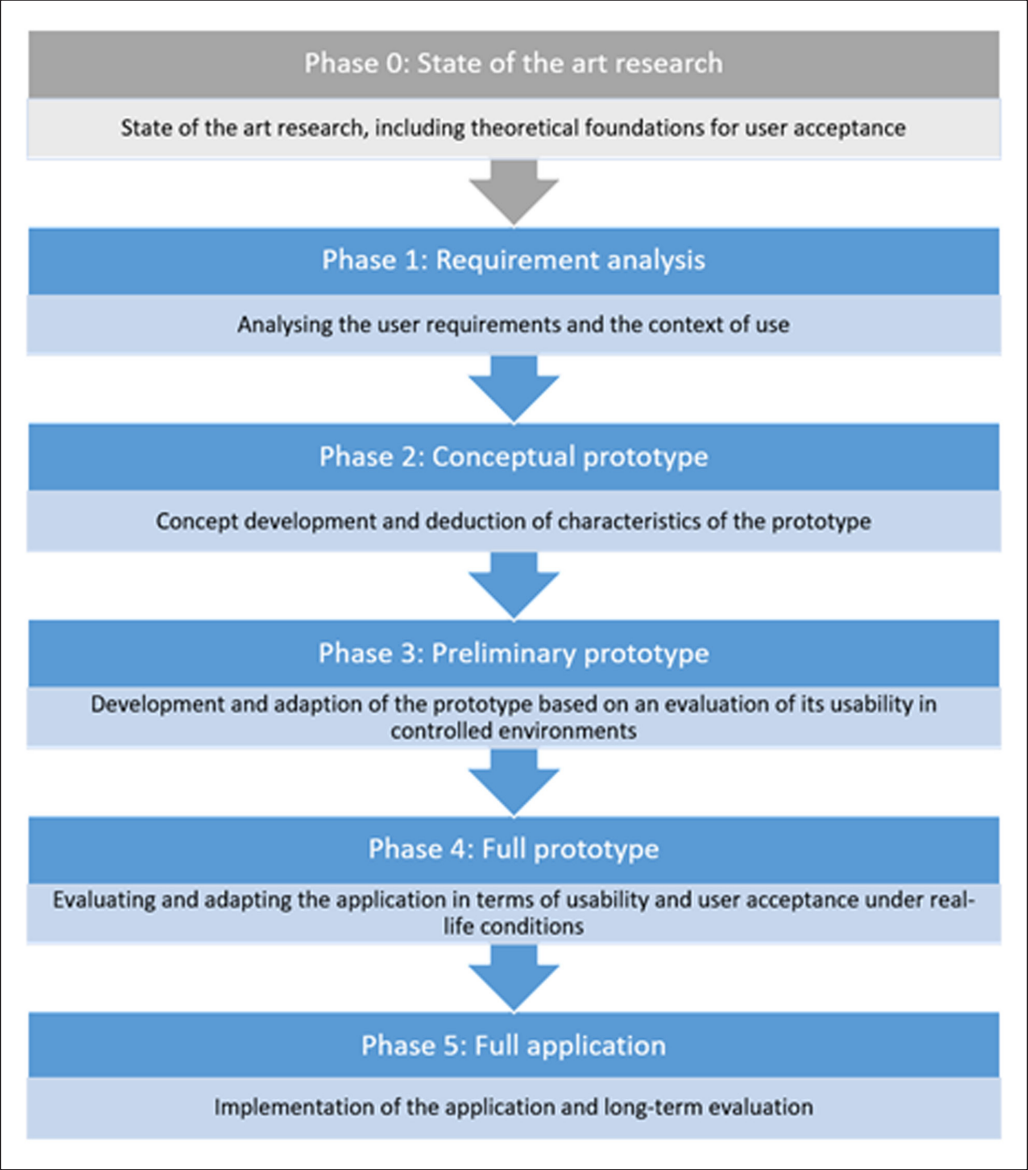
The phases of the generic blueprint for user-centred and participatory design processes.

Derived from 14 research items identified in the review process, the generic blueprint for user-centred and participatory design processes consists of six-phases and will be used as a canvas for the allocation of adequate methods. The allocation of each evaluation method to the phases is depicted in appendix 1. As a result of the review and qualitative alignment process, ***Figure 1*** depicts the different phases, including a short explanation of the tasks required in each phase.

Phase 0 covers relevant state of the art analyses, including theoretical basis, which should be considered during all consecutive steps. This *state of the art research* may comprise systematic or rather narrative review (e. g. desk research) methods [29] to identify guidelines, theories or other relevant projects to be used as a starting point. Phase 1, *requirement analysis*, is the pre-prototype phase, where user requirements, i. e. medical or professional needs and expectations towards a potential application, are gathered, preferably within the context the application will be used in. “Context” needs to be differentiated between the professional setting of health care providers and the social environment of patients.

The next phases constitute the prototype stages: In Phase 2, the results from Phases 0 and 1 are used to develop a *conceptual prototype* of the application, be it an actually usable prototype or just templates or sketches that depict the design (or main concept) of the application, including its layout. In Phase 3, the prototype is refined based on a first usability evaluation in controlled environments. Especially the user interaction with the prototype needs to be monitored and analysed leading to a *preliminary prototype*. This process is repeated in Phase 4 using an evaluation under real-life conditions with a *full-scale prototype* of the application, so that final changes can be made to improve ease of use and, therefore, user acceptance.

This concludes the prototype stages, so that finally, a *full-scale application* can be implemented and subjected to long-term evaluation in Phase 5. It is important to note that the process is iterative; meaning that in each phase the researchers and/or developers may take a step back to the previous phase if s/he thinks that important insights are missing.

The described prototypical participatory user-centred design process uses the background variables (input) defined by Esser & Goossens [25], including individual, organisational and technical contexts, to account for the complex interrelations of requirements healthcare IT has to deal with throughout the whole development process. Using these patient and context requirements to develop a patient-centred application that will be accepted by the patient is also supported by Arsand & Demiris [8]. The authors further call for context- and user-group-relevant methods being applied to support the iterative development of the prototype [8]. Finally, Sutcliffe & colleagues [31] argue that the evaluation of prototypes further helps to specify the volatile and complex requirements of the healthcare sector.

### Methods used for data collection in each Phase

Established evaluation methods are now presented with respect to their characteristics as a foundation for associating the appropriate methods to each phase. ***Table 1*** depicts the relation between the different process phases and the respective applicable methods.

**Table 1:** Applicability of methods for the different phases.


	COGNITIVE WALKTHROUGH	DESK RESEARCH	DIRECT OBSERVATIONS	EXPERT PANELS	FIELD INTERVIEWS	FIELD STUDIES	FOCUS GROUPS	HEURISTIC EVALUATIONS	IN-DEPTH INTERVIEWS	PAPER PROTOTYPING	PERSONAS	PROTOTYPING	QUANTITATIVE SURVEYS	QUANTITATIVE SURVEYS [USABILITY QUESTIONNAIRES]	SEMI-STRUCTURED INTERVIEWS	SEVERITY RANKING	APPLICATION SKETCHING	THINK ALOUD STUDIES	USE CASES	USER PROFILES	WORKSHOPS

0: State of the art research		x																			

1: Requirement analysis			x	x	x	x	x				x		x		x				x	x	x

2: Conceptual prototype							x			x	x	x			x		x		x		

3: Preliminary prototype	x							x	x					x		x		x			

4: Full prototype						x	x							x		x					

5: Full application					x	x									x	x					


Qualitative methods (e. g. semi-structured interviews and focus groups) are useful for gathering in-depth insights into user needs and expectations in Phase 1 [8]. They can be also used in the field, i. e. the natural environment of patients or the professional environment of health care providers in Phases 3 and 4 [32]. Such, they are ideal – albeit time-consuming – for understanding the context of use. Alternatively, quantitative surveys can be used for gathering the same information [33], yet without a deeper understanding of the users’ specific needs and expectations.

Existing personas may inform first needs for adaptation [34]. For developing life-like and application-specific personas, qualitative methods could be more apt [35], while user profiles can also be derived from cluster analysis based on survey data [36].

For the first prototype, testing it with reference to realistic use cases is essential. Discussing these use cases in focus groups or interviews can help to overcome the first hurdles that could hamper acceptance [37]. While actual working prototypes help to better simulate a usage scenario, paper prototypes or sketches are less cost-intensive alternatives [38]. In addition, changes in fully developed prototypes or systems are more labour-intensive as they might entail system-wide adaptions [39].

However, task-driven think-alouds [40] or cognitive walkthroughs [41] need to be conducted with a full-scale prototype in Phases 3 or 4. The same is true for quantitative system usability surveys as a more standardized alternative. Here, the IBM system usability scale, which measures satisfaction with the effort it took to achieve a task [42], is an apt choice. However, the more complex the system, the more usability issues can arise in the prototype phases. In order to prioritize challenges for the usability and the changes in the system they entail, a severity ranking can be used, ranging from “not a problem” (if a problem is only stated by a single user) to “usability catastrophe” (a problem that definitely has to be fixed before release of the application) [43].

### Use Cases demonstrating the applicability of the methodological guidance

Finally, in a third step, the applicability of the methodological guidance suggested is illustrated in three use cases highlighting the potential impact of working with the allocated methods in each phase of our generic blueprint process.

#### Use Case 1: Development and Testing of a Diabetes Self-Management App

The methodological guidance was applied in a study to develop the first diabetes self-management (DSM) app in Rwanda [44], called Kir’App. Diabetes self-management highly depends on feedback from various health care providers, e. g. primary physicians, diabetes educators or dietitians [9]. In this study, a mixed methods approach for the user-centred design of a diabetes self-management app was chosen. First, evidence for effectiveness of theory-based behaviour change interventions was gathered (Phase 0), suggesting that applications including features for patient-provider interaction and educative content are especially promising in terms of reductions in blood glucose levels [45].

Afterwards, 21 Rwandan patients with diabetes – as potential users of the envisaged app – were asked in semi-structured face-to-face interviews for their expectations towards a self-management app. The patients were contacted personally at the offices of the Rwandan diabetes association. Results of a qualitative content analysis of the interview transcripts indicate a need for diabetes-related knowledge, especially on how to conduct the daily life (nutrition and physical activity), monitoring of blood glucose values and reminders for medication intake as well as physical activity, and social support features such as means to contact other diabetics. In parallel, an analysis of local care structures and care practices, including diabetes-related health literacy, was conducted to explore unmet needs as relevant determinants of the context of use (Phase 1). As a result, a reliance on traditional medicine as a supplement to the existing healthcare systems in place was uncovered, as well as a lack of structured diabetes care programs [44].

**Figure 2:**
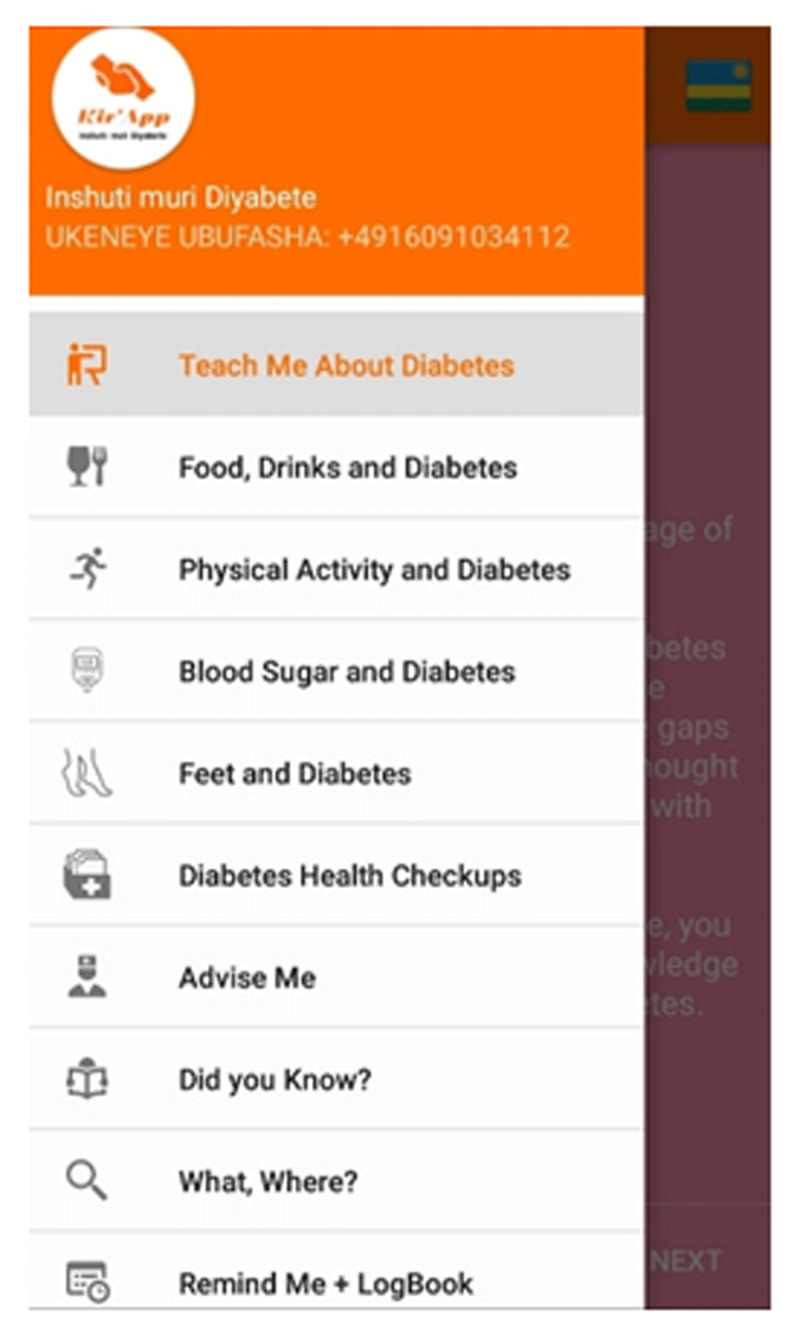
Functionalities of Kir’App.

Based on these qualitative insights, requirements for the mobile health app were categorized and a first prototype was developed, which contained the ten functionalities depicted in ***Figure 2***, ranging from information provision over a personal diabetes logbook to a reminder function (Phase 2).

In a next step, this prototype was made available to the same patients who were initially asked for their expectations. Fourteen of these chose to participate. This time, their experiences were collected after they used the app for three consecutive months. Again, semi-structured interviews were applied to assess whether the first prototype of the app met their overall needs and expectations. The categories gained in Phase 1 could all be identified again in the interview transcripts, showing that the prototype was in line with user expectations. Usability issues occurred e. g. when setting the alarm for medication reminders and could be sorted out afterwards. All in all, participants stated using the app increased their diabetes-related knowledge and aided in diabetes self-management [46]. In addition, a usability test based on the IBM system usability scale was conducted [42] (Phase 3). Preliminary results indicate that participants did not need exceptionally long to perform certain tasks and were satisfied both with the effort it took to achieve these tasks and with the overall usability of the app.

In a next step, a randomized controlled trial (RCT) will be conducted to evaluate the effectiveness of the app in terms of clinical outcomes (blood sugar levels; HbA1c) in Rwandan patients with diabetes (Phase 4). A focus group being carried out after or nested within the RCT will help to uncover specific functionalities of the application serving as drivers and barriers of sustained use, while also exploring factors affecting recruitment and implementation [47,48].

Evaluations of effectiveness will then be accompanied by a continuous assessment of user satisfaction, e. g. via an in-app questionnaire (Phase 5) [49]. The app is available in the Google Play Store and used by mainly Rwandan diabetics.

*Lessons learned*: For collecting user expectations towards a potential diabetes self-management application from diabetes patients, semi-structured interviews have proven especially valid because they provided a setting of confidentiality, so that knowledge gaps about the disease as well as difficulties with adhering to treatment regimes in everyday life could be uttered freely. Moreover, semi-structured interviews proved beneficial because they allowed for collecting heterogeneous – sometimes-conflicting – perspectives from a sample consisting of patients with heterogeneous levels of health literacy and experience with digital technologies. The literature research warranted in Phase 0 was essential to understand the cultural background of the participants in the interview sessions.

#### Use Case 2: A discharge management online platform

The number of beds per capita as well as lengths of hospital stays are continuously reduced in most European countries [50]. The latter is regarded as a measure of efficiency [51]. Although shorter stays are intended to reduce costs by moving patient care from the highly service intensive and costly inpatient care setting to ambulatory care, this requires intensive aftercare coordination during the short hospital stays [52]. Based on the methodological guidance, a discharge management online platform was developed by a German statutory insurance company in collaboration with a university clinic to facilitate this organizational process.

In Phase 0, state of the art research indicated that discharges from hospitals are prone to care fragmentation and losses of patient information and may lead to post-discharge readmissions [53]. The competing medical and social demands of chronic care patients urge for a structured, timely and medically acceptable coordination of the hospital discharge and care transition process without putting the patient at risk [53]. Digital discharge management strategies need to support nurses and other personnel in coordinating relevant post-acute services and remedies as well as to apply for funding of these services with the health insurers [54]. However, no digital device of this configuration exists to date.

Therefore, semi-structured interviews with nurses and other personnel responsible for the discharge management were conducted to determine possible aspects of the application process to be improved by an online platform (Phase 1). The relevant personnel was recruited via E-Mail and in recurring meetings by the head of the hospital social service units. The results show that the personnel involved wished for an online application that improves knowledge of a patient’s situation (concerning e. g. her/his residential situation as well as assistance systems already in place) early after admission. The latter would save time in the discharge process, guaranteeing earlier discharge. Furthermore, the personnel interviewed whished for an uplink to the existing hospital information system, paperless processes, including an option for digital signatures, and an intuitive user interface. Above all, they were adamantly opposed to a system which would only be applicable for interacting with one instead of all insurance agencies, as this would lead to extra work.

The first software prototype (Phase 2) was subject to a technical testing before nurses, case managers and social service personnel at the university clinic were trained in two sessions in using the software interface. The training sessions were observed openly by two researchers (field study). Questions arising in the sessions were collected and used to re-adjust the interface (Phase 3). For example, during the training sessions, the participants expressed a need to be able to proceed through the systems without knowing the exact ICD-10 diagnosis code, and to upload necessary documents days after first dealing with a patient within the system.

After a first live testing phase (Phase 3) of four weeks in orthopaedics and trauma surgery wards, technical issues had to be resolved before the online platform was used in almost all wards equipped with patient beds.

Although focus groups were preferred to engage discussion among the users of the online platform for the next evaluation step (Phase 4), restrictions due to COVID-19 caused a switch to an online semi-standardised survey. Focal topics of the online survey included an assessment of users (including technical affinity and work experience in years) and usage of the online platform measured in days. Afterwards, participants were asked to list problems in their line of work, focussing on the period before using the online platform. The subsequent questions focussed on menu navigation and general design of the app. The integration of the system into everyday clinical practice in the wards and its usefulness when dealing with complex cases, e. g. patients with multiple chronic conditions, was subject of further questions. The system usability scale, a standardised 10-item questionnaire [55], was used to measure user satisfaction and ease of use. Usability testing also included several use cases depicted in screenshots of the interface as part of the survey.

Finally, participants were asked to describe their wishes for a “perfect online platform in discharge management”. Consequently, the mapping of a) problems in the everyday line of work of discharge managers, b) strengths and c) weaknesses of the online platform as well as d) reflections on a “perfect online platform” from a user perspective are expected to pave the way for additional functions and updated versions of the online platform, which is currently in re-development mode.

After finalising the full application, economic analyses will be conducted to evaluate changes in terms of length of stay or 30-day readmission rates. These analyses will be accompanied by continuous assessments of perceived usefulness by the nurses.

*Lessons learned*: The initial semi-structured interviews proved to be a time-saving way to gather initial expectations towards the discharge management system. However, they also showed that participants had difficulties envisioning such a system. Therefore, the field study during the training session with the first functional prototype yielded more practical feedback on how to improve the system. The same is true for the semi-structured online questionnaire, which also allowed for linking quantitative assessments of usability with qualitative insights on the system as a whole, showing that missing functionalities outweigh an overall good usability. It also allowed to gain insights from case managers working in different medical disciplines and into corresponding requirements towards the new system.

#### Use Case 3: Assessment of an Automated Design Process for customized Health Smart Homes

In contrast to individual digital health applications, Ambient Assisted Living (AAL) or Health Smart Home (HSH) systems [56] are complex multi-component-based systems that are composed of already existing applications and products [57]. Integrated care in the context of AAL therefore refers to integrating a set of individual monitoring and assistance tools to aid chronically ill patients [58]. In this case, customization in a user-centred design process is achieved by an appropriate selection and integration of components-off-the-shelf, without the need for development or alterations of the individual products themselves. The appropriateness of components is mainly determined with regard to the users’ needs, which are motivated by the users’ medical conditions; however, general user preferences can also be taken into account [59].

**Figure 3:**
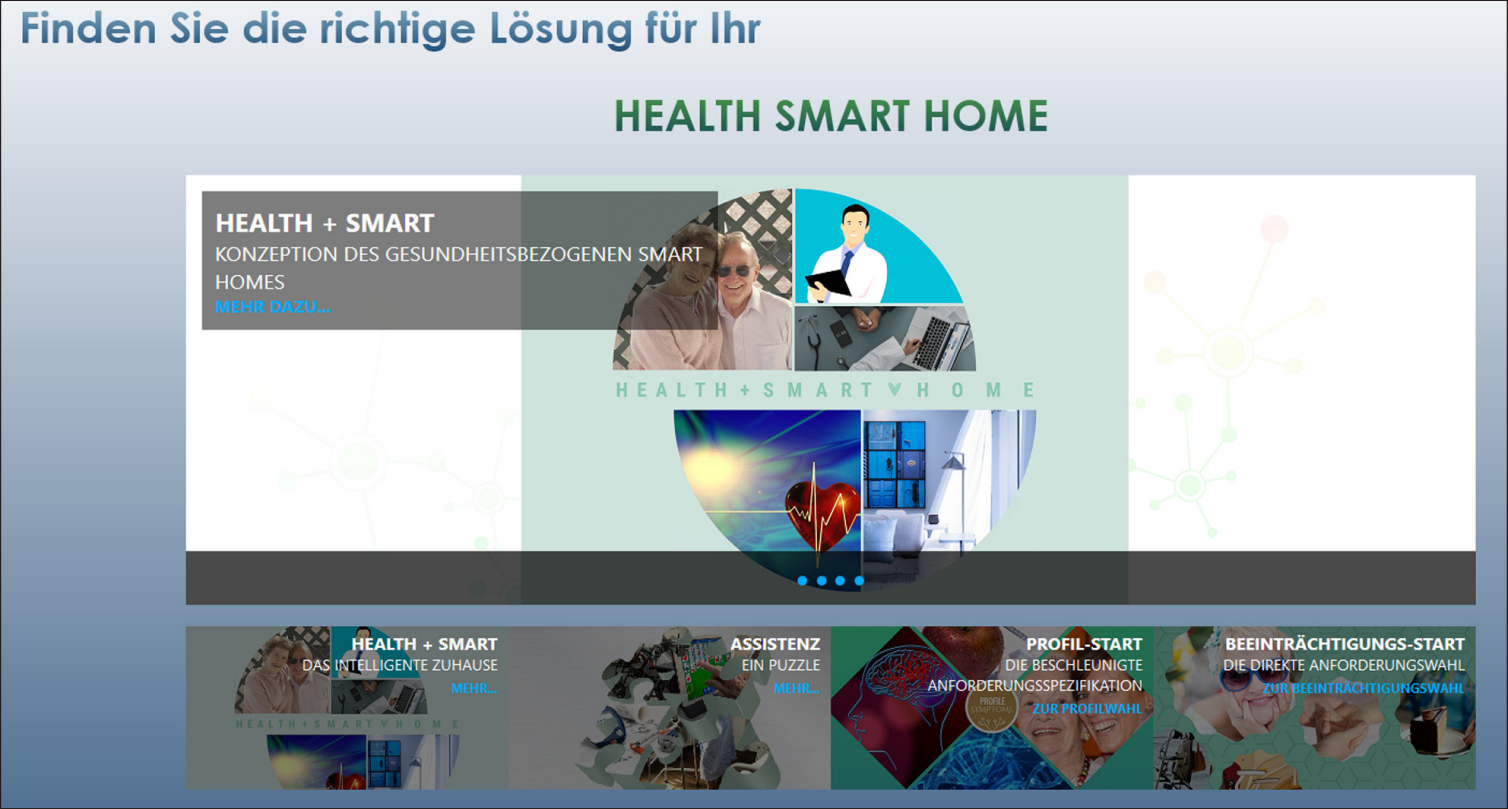
Start screen of the web application for suggesting need-based assistance systems inviting users to find the right solution for their Health Smart Home. In the lower part, information on health samrt homes can be procurred and different options for interacting with the system are offered.

Since it offers an orthogonal approach of customization, this domain is suitable to demonstrate the broad applicability of the presented methodological guidance. Therefore, this use case will discuss the application of the proposed methodological guidance in developing an automated AAL suggestion system. This AAL suggestion system has been proposed on a theoretical level by Wollschlaeger and Kabitzsch [60], and facilitates the design and tailoring process for AAL solutions during patient consultation. AAL consultation is a two-tier process, consisting of the actual consultation and subsequent follow-up evaluations. Addressing the consultation activities, the automated AAL suggestion system is intended to be used by AAL counsellors in an interview-style setting with a patient, yielding possible and need-based system candidates. A prototype of the suggestion system has been implemented as a web-based application (cf. ***Figure 3***) and facilitates a participatory approach to composition and tailoring of component-based systems. Thus, the actual artefact that is being developed using a participatory design approach is the customized and need-based Health Smart Home.

As a consequence, the guidance was not primarily used for developing the AAL suggestion system itself, but it rather provided orientation on how the AAL suggestion system should facilitate the consultation process.

The web-based application starts with an analysis of the user requirements (Phase 1). These requirements are subsequently used to determine appropriate abstract assistance concepts, tailored to the respective user (Phase 2). Finally, suitable assistance components implementing the required assistance concepts are selected from a database and suggested to the AAL counsellor and the patient. At the end of the consultation, a customized AAL system can be selected based on these suggestions and installed at the patient’s home (Phase 3).

The different steps provided by the described AAL suggestion system are in accordance with the blueprint and the provided methodological guidance. On the one hand, this provides guidance and justification for the early Phases 1 to 3. On the other hand, suitable evaluation methods that are valuable for designing the organisational framework for the suggestion system (Phases 3 to 5) can be identified from the proposed method set.

**Figure 4:**
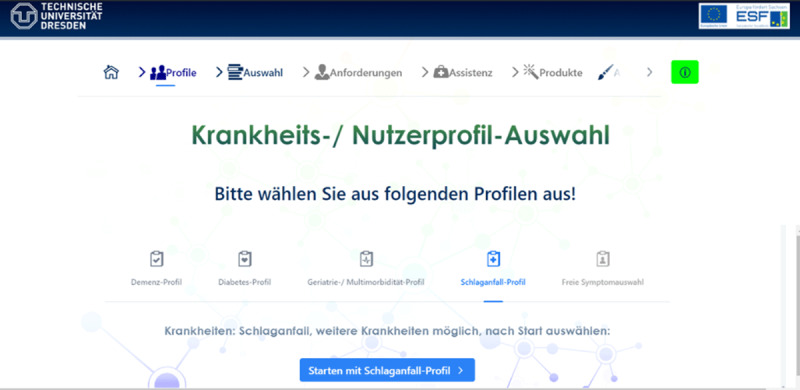
Screen for starting with a pre-defined patient profile. Profiles to be chosen are dementia, diabtes geriatrics/multi-morbidity, stroke, and free selection of symptoms.

More specifically, in Phase 1, the user requirements are analysed based on a standardized questionnaire, which has been developed according to personas, user profiles of patients and insights of health care providers (cf. ***Figure 4***). Both the use of pre-defined profiles, including dementia, diabetes, geriatrics, or stroke, and a custom configuration of user requirements is supported by the AAL suggestion system.

**Figure 5:**
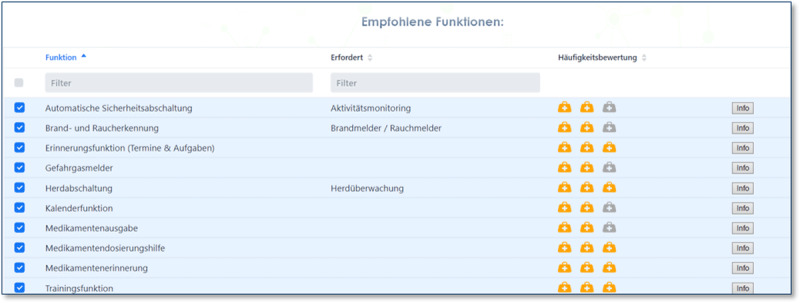
The suggested assistance concepts of the consultation web app are ranked according to their usefulness for the chosen profile.

The concept development of Phase 2 is realized by suggesting appropriate assistance concepts on an abstract functional level (cf. ***Figure 5***), thereby sketching the intended applications and providing a rough prototype. In the presented example, the suggested assistance concepts include automated equipment deactivation, detection of fire and smoke, reminder & calendar functionalities, and medication support.

**Figure 6:**
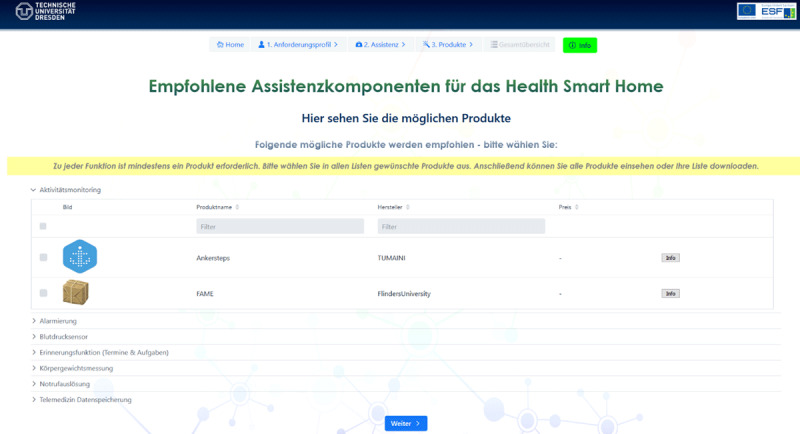
At the end, assistance products are proposed for each suggested assistance concept.

The subsequent suggestion of specific assistance products provides input for the development of a specific prototype in Phase 3 (cf. ***Figure 6***). For each assistance concept identified in the previous step, suitable assistance products are proposed. As can be seen from ***Figure 6***, the functionality “activity monitoring” can be provided by the step counters connected to mobile apps such as *my sugar*, which is an app for diabetes prevention [61], or FAME, an app developed for patients with early-stage dementia [62].

As a consequence, the AAL suggestion system is a tool offering support for the first steps of a user-centred design process.

The further evaluation actions of Phases 3 to 5 address the organisational context of the suggestion system. Thus, the method set proposed by the methodological guidance introduced in this article provides guidance on how to design evaluation steps and follow-up evaluations. It can be inferred from the description of Phase 3 that shortly after installing the assistance components in the user’s home, a follow-up evaluation regarding usability is warranted to make sure the user can operate the components as intended. Based on the method set for Phase 3, interviews, think-aloud studies, and usability surveys as described in use case 1 could be appropriate tools for this evaluation. After this first tutorial phase, the user should be familiar with the new components and their use. It is now possible to assess the efficacy and user acceptance in real-life conditions using field observations and interviews (Phase 4). Finally, the long-term effects on the patient’s quality of life or her/his coping with the disease, as well as possible need for adaptations can be evaluated using semi-structured interviews or quantitative surveys with the patient, the AAL counsellor, and the patient’s general practitioner (Phase 5). At any given time, a new iteration of the consultation process can be started, as suggested by the blueprint in section 5.1.

A prototype of the suggestion system was implemented and evaluated by both case managers and patients. Further development and introducing support for the last phases of the design process as well as the follow-up evaluations is a currently ongoing activity in order to enhance the applicability of the suggestion system [60].

*Lessons learned*: In this use case, the developed AAL suggestion system itself is used as a facilitator for a user-centred and participatory selection and composition of assistance technology for patients requiring technical assistance in their homes. During the development of the suggestion system, the evaluation guide proved beneficial for selecting possible interaction patterns with the users. Thus, a questionnaire setting combined with personas and exemplary user profiles could be suggested as suitable for analysing the patients’ requirements. Initial test users of the prototype of the AAL suggestion system (medical experts such as case managers and practitioners, but also potential patients and their relatives) confirmed this setting to be a viable approach for the analysis of requirements. Instead of presenting the suggested assistance components straight after this analysis, we consulted the guide and chose to present intermediate results (in terms of neutral assistance functionalities) as a kind of application sketch. The feedback provided by the initial test users indicates that this step-by-step approach facilitates a deeper understanding of the proposed assistance components by the patients.

As we are fully aware that the decision support offered by the AAL suggestion system calls for follow-up evaluations, we identified the proposed evaluation methods of Phases 3 to 5 as helpful input when designing the organisational context the suggestion system should be used in. Yet, further practical application of the suggestion system is required in order to assess how beneficial the contributions of the evaluation guide for the organisational context design actually was.

## Discussion

### Summary of results

Using the phases of a generic blueprint for user-centred and participatory design processes of health care IT as a starting point, we provided adequate methodological guidance as a scientific foundation for the participatory development process of healthcare IT applications. This, in turn, limits the risk that the success of applications is not hampered by its non-adoption by the prospective users [2]. Our three use cases demonstrate the applicability of our proposed methods and the phases to which they are assigned for developing a self-management app from scratch and without previous knowledge of the user group, but also for building a discharge management platform in a hospital setting and for combining existing AAL and HSH solutions.

The application of the blueprint, enhanced with established scientific methods of evaluation, enables health care IT developers to address the complex requirements of healthcare IT mentioned in the beginning. Following the methodological guidance for each generic phase helps capturing the interrelations between heterogeneous patient characteristics (due to age, health literacy, individual health care preferences, previous experiences with technology, and beliefs of self-efficacy [16]), the communication between and cooperation of various stakeholders, and health care system requirements relevant to reimbursement of health services. As such, the generic nature of our methodological guidance may support especially healthcare IT developers with less experience in evidence-based evaluations of healthcare services to adapt and apply our recommendations to heterogeneous use cases and settings in a step-wise manner.

### Comparison to prior research

A recent analysis revealed a wide range of definitions and applications of relevant terms like acceptability, acceptance and adoption [63]. Our generic blueprint for user-centred and participatory design processes, being accompanied by established methods for evaluation, may also contribute to an improved level of standardisation. In general, our process is in line with existing practices for user-centred design as they are made publically available, e. g. by the platform “usability.gov” [64]. It also corresponds to the principles of Cooperative Design, as we aim for the participation of all relevant stakeholders in the design process [65]. However, our approach necessarily goes beyond the existing ones: With respect to digital health applications used in the integrated cares setting, the patient-provider interaction as well as the interaction between providers needs to be in the focus of all six steps. Theoretical frameworks for the integrated care of the chronically ill, such as the Chronic Care Model [66], should drive the development of the first prototype [67]. The needs assessment should also cover the requirements of patient-provider- and provider-provider communication [68]. As for the full-scale implementation and continuous evaluation of healthcare IT applications in an integrated care setting, changes in the interaction, in particularly the shared decision making process, need to be monitored [69]. However, patient-provider interaction remains unaccounted for in several implementation processes for integrated care systems, e. g. for the Central Coast Integrated Care Program [70].

In addition, a clear assignment of adequate methods to each consolidated step has not been provided so far. Consequently, analyses of established European integrated care programs targeting patients with multi-morbid conditions show that these are insufficiently equipped with adequate methods to support patient involvement [71]. Our research contributes to closing this gap and thereby helps decreasing the risk of methodologically flawed evaluation studies [72], where, e. g., Patient-Reported Experience Measures are disregarded [73].

As the development of user-centred interventions and application systems is a complex phenomenon, we take into account not only the individual him-/herself, but also his/her context of use, which is represented, e. g., by his/her conduct of daily life and disease coping mechanisms [74]. Whether initial expectations have been met needs to be considered before roll-out of any technological innovation [19]. Therefore, our several iterative steps (Phases 2 to 5) are incremental and in line with the PDCA cycle [75] as well as the established key elements of integrated care [76]. The idea of conceptualising product development levels and evaluating the maturity of the product accordingly is the focus of the established technology readiness assessment [77], as well as an essential part of Roger’s Diffusion of Innovations theory [78]. Our proposal puts a stronger emphasis on individual and contextual characteristics [79] and how they can be captured best with scientific evaluation methods. As such, it is likely that an integration of our methodological guidance into existing procedures, like models frequently applied in software development projects (e. g. spiral models [80], V-model or agile approaches like Scrum [81]; for a conclusive overview see [82]) may have an additional benefit, especially since those often lack an evidence base. As we aim to provide methodologies for co-creating healthcare IT applications, they fit very well into existing living lab approaches [83]. An early consideration of the end user, i. e. his/her needs and expectations, is one of the methodological requirements according to the newly developed NICE framework for digital health evaluation. Levels of evidence stratified by functional classifications as introduced by the NICE framework may further guide the evaluation and certification process in all of our use cases [84].

Our approach puts an emphasis on methods that allow user participation throughout the whole process. Such, it promotes a shift from user-centred to participatory design [23].

From a methodological standpoint, we provide methods from both the qualitative and quantitative realm, which is in line with the notion that mixed methods might be best suited to capture the complex requirements of implementation of novel techniques in health care [85]. While the methods we propose are not new themselves, we demonstrate how they can be adapted and combined to serve the complex requirements posed by the health care sector (see introduction).

### Limitations

Although our proposal is based on recent evidence in the field of healthcare IT, our approach has limitations. First, no criteria-driven inclusion process was carried out to identify and include relevant evidence. As we did not aim at developing an all new user-centred design framework, but rather aimed at collecting methods to be applied to ensure user participation in each step of a generic blueprint, we found a literature review [29] to be sufficient. However, a broader set of databases, such as, Embase Web of Science or Scopus, could have yielded more results. As such, we may have missed relevant manuscripts and the identified methods corresponding to the phases may not be exhaustive. One example is the consideration of adequate study designs in Phases 4 and 5. It is recommendable to choose study designs that correspond to the types and functionalities of the innovation and simultaneously consider the maturity and efficacy of the prototypes.

As a common limitation of qualitative studies, the individual backgrounds and subconscious expectations of the authors may have also had an influence on the consolidation of phases and the mapping of methods. As such, the suitability of the proposed methodological guidance for providing orientation in selecting adequate methods during the development process requires further validation, especially concerning its Phases 4 and 5. Although the presented use cases allow for illustrating the applicability of the derived methodological guidance across different healthcare IT phenotypes (i. e., self-management app, decision-support system) and target audiences (i. e., patients, case managers), they were selected purposefully and are therefore prone to subjectivity.

### Future research to validate our methodological guidance

For this purpose, major evaluation studies comparing our approach with others head to head do not seem realistic. Instead, alternative means for validation are required. One possibility would be to have different projects carry out a user-centred design with different process models. These real-world projects could be scientifically accompanied in order to evaluate the extent of user participation and the usability of developed digital health solutions depending on the chosen process model.

## Conclusions

With the introduced methodological guidance, we provide methodological guidance enabling technicians, health care professionals and other stakeholders interested in healthcare IT, to develop user-centred digital health interventions systematically. Applicants of our methodological guidance can make use of the guiding phases and the associated methods to assess and verify design decisions and iteratively evaluate their own intervention or product. The use cases presented highlight the utility of the blueprint as well as the methods assigned to its phases.

Scientifically monitored utilisation of our guidance in diverse settings may contribute to continuously improve its compatibility with the requirements of healthcare IT developers and the alignment with existing process models. Future research is needed to systematically enrich and prioritise the suggested methods and validate the proposed methodological guidance.

## Appendix

**Appendix 1:** Allocation of Phases, suggested methods and the corresponding research items.

PHASE	SUGGESTED NAME OF PHASE/OBJECTIVE	SUITABLE METHODS OR INSTRUMENTS

**0**	*State of the art research, including theoretical foundations for user acceptance* [35,44]	*Desk research*

**1**	*Analysing the user requirements* [8,32,35,37,38,86,87,88,89,90,91] *and the context of use* [92,93]	Direct observations [32,89] Use cases [20,35,38] Expert panels of patients and health care providers [90] Workshops with patients [93] Semi-structured and field interviews with patients [35,37,38,88,89,90], their relatives, health care providers [32,35,37,38] and hospital managers [37] Quantitative surveys with patients [20,91] Focus groups with patients [32,38,87] and health care providers [87,88,93] Personas and user profiles of patients [20,32,35,86] and health care providers [35,86] Field studies [32,88]

**2**	*Concept development* [38] *and deduction of characteristics of the prototype* [37,86,87,88,89,91,92] *based on an in-depth understanding of the user requirements* [8,32,35,86,88,90,92,93] *and context of use* [8,90]	Use cases [38] Focus groups with patients [38] and health care providers [37] Semi-structured interviews with patients [38,88] and health care providers [38] Paper Prototyping or Sketching of the intended applications [20,35,88,90] Personas of patients [90] Prototyping [32,35,86,87,88,89]

**3**	*Development* [8,32,35,38,86,87,89,90,92,93] *and adaption* [32,86,87] *of the prototype based on a first evaluation* [8,20,35,37,86,87,88,89,91,92] *of its usability in controlled environments*	Cognitive walkthrough with health care providers [20] Heuristic evaluation with non-users [20,93] Think aloud studies with health care providers [20,88,92], patients [88,92,93] and their relatives [89] Interview [87,89,90] Quantitative surveys with patients [20,37,88,90,91,93] and health care providers [20,87,88], using usability questionnaires

**4**	*Evaluating* [8,32,37,38,86,89,93] *and adapting* [37,91,93] *the application in terms of usability and user acceptance under real-life conditions*	Field studies [88] Focus groups with health care providers [37] Quantitative surveys with patients [20,37,88,90,91,93] and health care providers [20,88], using usability questionnaires

**5**	*Implementation* [32,35,37,91,92] *of the full-scale application and long-term evaluation* [35,37,90,93]	Semi-structured interview with patients [38,92] and health care providers [92]
